# Desmoid tumors of rectus abdominis: A case report and literature review

**DOI:** 10.1097/MD.0000000000039089

**Published:** 2024-07-26

**Authors:** Hong-Peng Guo, He Zhang, You Li, Xing-He Pan, Cheng-Lin Sun, Jun-Jie Zhang

**Affiliations:** a Department of General Surgery, Central Hospital of Shenyang Sujiatun, Shenyang, China; b Department of Orthopedics Surgery, Central Hospital Affiliated to Shenyang Medical College, Shenyang, China; c Department of General Surgery, Central Hospital Affiliated to Shenyang Medical College, Shenyang, China; d Department of Pathology, Central Hospital Affiliated to Shenyang Medical College, Shenyang, China.

**Keywords:** abdominal, active surveillance, *CTNNB1*, desmoid tumor, surgery

## Abstract

**Rationale:**

Desmoid tumor (DT) is a rare soft tissue tumor that can occur anywhere in the body. Abdominal wall DT presents unique clinical challenges due to its distinctive manifestations, treatment modalities, and the lack of biomarkers for diagnosis and recurrence prediction, making clinical decisions exceedingly complex.

**Patient concerns:**

A 32-year-old female who underwent radical resection combined with patch reinforcement for rectus abdominis DT, successfully alleviating abdominal discomfort, with no recurrence during the 6-month follow-up after surgery.

**Diagnoses:**

Based on the imaging studies and medical history, the patient underwent radical surgical resection. Histopathology reveals that the tumor cells predominantly composed of proliferative fibroblasts with local collagen deposition. The lesional cells show positive staining for β-catenin, indicating a diagnosis of DT.

**Interventions:**

The patient underwent radical surgical resection with patch reinforcement to repair the abdominal wall defect. Pathology confirmed negative margins, achieving an R0 resection, and genetic testing identified a *T41A* mutation in *CTNNB1*. Consequently, no additional adjuvant therapy was administered postoperatively.

**Outcomes:**

The patient was discharged with the incision healing well after 3 days postoperation. Upon reexamination 6 months later, no recurrence or adverse complications were observed.

**Lessons:**

Abdominal wall DT treatment requires personalized plans from multidisciplinary team discussions. Genetic testing plays a crucial role in identifying novel biomarkers for abdominal wall DT. We have once again demonstrated the significant clinical significance of *CTNNB1* mutations in the diagnosis and progression of abdominal wall DT. Additionally, genes such as *CCND1, CYP3A4, SLIT1, RRM1, STIM1, ESR2, UGT1A1*, among others, may also be closely associated with the progression of abdominal wall DT. Future research should delve deeper into and systematically evaluate the precise impact of these genetic mutations on treatment selection and prognosis for abdominal wall DT, in order to better guide patient management and treatment decisions.

## 1. Introduction

Desmoid tumor (DT), also known as desmoid fibromatosis (DF), or aggressive fibromatosis (AF), is a rare clonal proliferative tumor originating from deep soft tissue.^[1,2]^ The incidence of DT is extremely low, with an annual rate of 2 to 4 cases per million individuals, accounting for approximately 0.03% of all tumors. Between 3.5% and 32% of cases are associated with familial adenomatous polyposis (FAP) or Gardner syndrome.^[3]^ The growth pattern of DT is variable; it may gradually increase, remain stable, or spontaneously regress in some instances.^[4,5]^ Although significant progress has been made in DT research over the past decades, the rarity of DT results in limited related case reports and treatment experiences. Specifically, the unique clinical manifestations and treatment challenges of abdominal wall DT further complicate clinical decision-making.^[6,7]^ Therefore, personalized treatment plans should be developed based on the specific circumstances of the patient within a multidisciplinary team (MDT) diagnostic and treatment model.

Here, we report a case of progressive enlargement of the abdominal wall DT. The patient’s abdominal discomfort was successfully alleviated by radical resection combined with patch reinforcement to repair the abdominal wall defect. Subsequently, comprehensive examinations, including immunohistochemistry and gene testing, were performed on the pathological tissues. This article aims to discuss the clinical and pathological characteristics, as well as the diagnostic and treatment strategies of this case, along with a review of relevant literature.

## 2. Case report

**Figure 1.**
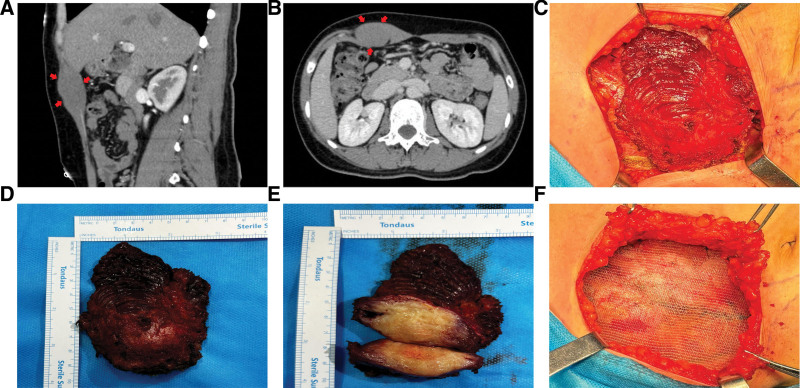
A 32-year-old female presented with DT of the right rectus abdominis, showing preoperative CT scans (A, B), surgical resection of the tumor (C–E), and patch reinforcement for repairing the abdominal wall (F). DT = desmoid tumor.

A 32-year-old woman observed a painless, fixed mass on her right abdominal wall, approximately the size of an egg yolk, 1 year ago. This untreated mass gradually enlarged and became painful, prompting her to seek medical attention at our institution. Three years earlier, she had undergone a successful cesarean delivery and denied any other history of surgical interventions, trauma, or familial genetic diseases. Physical examination revealed a hard, well-defined, immobile mass in the right upper quadrant, which was tender upon palpation. Ultrasound identified a 3.6 × 2.2 × 6.6 cm oval, hypoechoic region within the right upper abdominal musculature, indicative of encapsulation with prominent vascular flow. Enhanced abdominal CT depicted a round, iso-dense mass measuring approximately 2.2 × 3.7 cm along the right rectus abdominis, with well-defined margins and CT values of 39 HU, increasing to 45 HU in the arterial phase and 66 HU in the venous phase, demonstrating vascular enhancement (Fig. 1A and B). Laboratory tests, including tumor markers, were within normal limits.

Following a comprehensive evaluation by a MDT, it was noted that the patient’s rectus abdominis mass had recently increased in size and was causing abdominal pain. The patient declined further observation and explicitly requested surgical intervention. The surgery was conducted under general anesthesia through a 15 cm incision over the right rectus abdominis. Sequentially, the skin, subcutaneous tissue, and anterior sheath of the rectus musculature were incised. Upon exploration, the tumor was found to be completely encapsulated by the rectus muscle. The muscle and its sheath from the upper right abdomen were excised down to 10 cm in length and 8 cm in width (Fig. 1C). The tumor, along with part of the rectus muscle, some posterior sheath, and the outer margin of the external oblique aponeurosis, was completely excised. The tumor’s cut surface appeared gray-white and was firm in texture (Fig. 1D and E). Intraoperative frozen pathology suggest a possible diagnosis of DT, with tumor-free margins. After achieving hemostasis, the posterior sheath was sutured with absorbable sutures. Sufficient space was created anterior to the sheath to accommodate a 15 × 10 cm self-adhesive patch (Fig. 1F), which was secured to the anterior sheath with absorbable sutures. The layers were then closed in sequence, following verification of the correct count of gauze and instruments.

**Figure 2.**
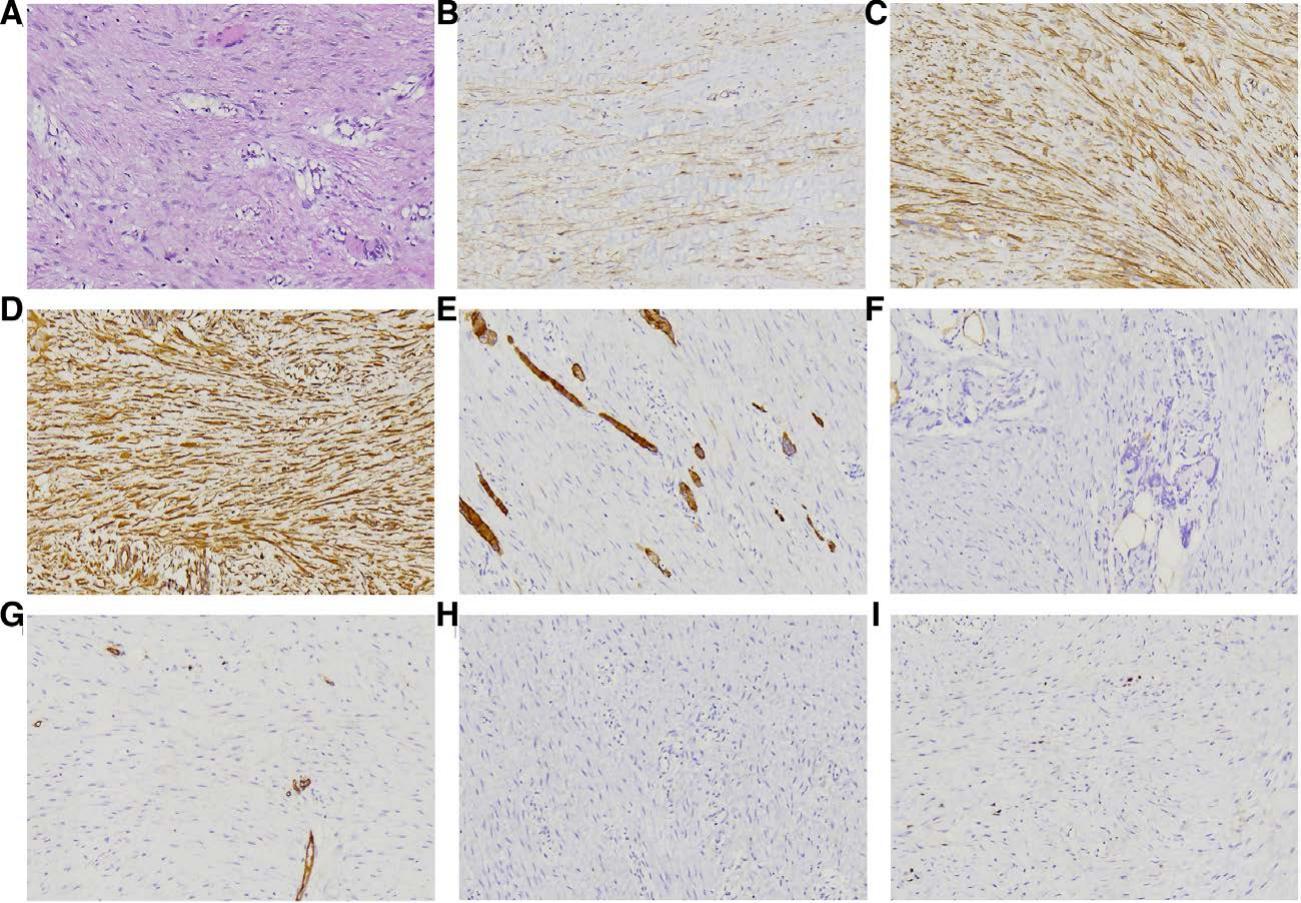
Representative photomicrographs for pathologic diagnosis. (A) Microscopic view showing the tumor predominantly consists of proliferative fibroblasts (H&E, 200×). (B) Immunohistochemical analysis positive for β-catenin (200×). (C) Immunohistochemical analysis positive for SMA (200×). (D) Immunohistochemical analysis positive for vimentin (200×). (E) Immunohistochemical analysis negative for desmin (200×). (F) Immunohistochemical analysis negative for S-100 (200×). (G) Immunohistochemical analysis negative for CD34 (200×). (H) Immunohistochemical analysis negative for CK (200×). (I) Tumor cells showing a very low Ki-67 labeling index (200×).

Gross examination of the specimen revealed dimensions of 5 × 4.8 × 2.8 cm with a gray-yellow cut surface and a firm consistency. Hematoxylin and eosin (HE) staining of the paraffin-embedded tissue sections revealed tumor cells predominantly composed of proliferative fibroblasts with local collagen deposition. Additionally, uniformly distributed thin-walled blood vessels were interspersed with localized areas of hemorrhage and lymphocytic infiltration. The fibroblasts were spindle-shaped, mild in appearance, arranged in parallel or wavy patterns, with sparse or vacuolated chromatin and 1 to 2 visible nucleoli. Mitotic figures were either absent or rare. The tumor exhibited unclear margins and invasive growth, with tumor cells infiltrating surrounding adipose tissue and striated muscle. Atrophy was observed in some striated muscle along with multinucleated myocyte giant cells (Fig. 2A). Immunohistochemical analyses were positive for β-catenin (Fig. 2B), SMA (Fig. 2C), and vimentin (Fig. 2D), whereas Desmin (Fig. 2E), S-100 (Fig. 2F), CD34 (Fig. 2G), and CK (Fig. 2H) was negative. Approximately 1% of cells were Ki-67 positive (Fig. 2I). Genetic testing analysis, including 198 genes such as gene mutations, gene copy number variations, and gene fusions, revealed a positive *CTNNB1* mutation (c.121A > G, p.Thr41Ala) and negative *APC* mutation. Results of other gene that tested positive are listed in Table 1.These examination findings confirm the diagnosis of DT.

**Table 1:** Genetic testing results.

Gene	Test Content	Test Result	Region	Specific Result	Specific Value
CTNNB1	Mutation	Positive	E3	c.121A > G,p.Thr41Ala	18.5%
CYP3A5	Mutation	Positive	Intronic	c.219-237G > A	46.6%
VEGFA	Mutation	Positive	5′UTR	c.-2055A > C	25.8%
VEGFA	Mutation	Positive	5′UTR	c.-958C > T	41.2%
VEGFA	Mutation	Positive	5′UTR	c.-94C > G	45.5%
VEGFR2	Mutation	Positive	E11	c.1416A > T	42.5%
CCND1	Mutation	Positive	E4	c.723G > A	100%
NQO1	Mutation	Positive	E6	c.559C > T	38.8%
RRM1	Mutation	Positive	3′UTR	c.*151A > T	98.1%
STIM1	Mutation	Positive	Intronic	c.1138-52A > C	99.5%
STIM1	Mutation	Positive	3′UTR	c.*367G > A	99.7%
AXIN2	Mutation	Positive	Intronic	c.1060-77G > T	46.1%
ESR2	Mutation	Positive	Intronic	c.1406 + 1643A > G	99.4%
CBR3	Mutation	Positive	E1	c.11G > A	51.1%
CYP3A4	Mutation	Positive	Upstream	c.-392G > A	100%
XRCC1	Mutation	Positive	E10	c.1196A > G	46.9%
SLIT1	Mutation	Positive	Intronic	c.198-4295T > C	100%
C8orf34	Mutation	Positive	Intronic	c.736 + 8162C > G	46.6%
UGT1A1	Mutation	Positive	5′UTR	*28	89.3%
UGT1A1	Mutation	Positive	5′UTR	c.-3152G > A	99.4%
MTHFR	Mutation	Positive	E5	c.665C > T	45.6%
MTRR	Mutation	Positive	E2	c.147A > G	50.9%
ESR1	Mutation	Positive	Intronic	c.1369 + 123G > A	42.4%
XPC	Mutation	Positive	E16	c.2815C > A	44.3%
CYP2C8	Mutation	Positive	Intronic	c.1291 + 106G > A	35.6%
CYP2C19	Mutation	Positive	E5	c.681G > A	28.4%
CYP2D6	Mutation	Positive	E6	c.886T > C	46.7%

The patient was discharged 3 days post-surgery with a good recovery. Follow-up visits showed no complications, and no additional treatments were administered. Six months later, a follow-up visit and colonoscopy revealed no signs of recurrence or FAP.

## 3. Discussion

DT is primarily sporadic, accounting for 85% to 90% of cases, and is associated with various factors such as trauma, pregnancy, and hormonal fluctuations.^[8]^ We reviewed cases of abdominal wall DT reported from 2021 to 2024 (Table 2).^[9,21]^ In these 14 cases, the ages varied from the 20s to the 60s, with a significant predominance of female patients. Most patients had a history of surgery or trauma, with a particular prevalence among female patients, including 4 cases of cesarean sections. The case we report also had a history of cesarean section 3 years earlier. This indicates that abdominal wall stress and cesarean sections during or following pregnancy may contribute to the development of abdominal wall DT. Additionally, the occurrence of DT may be related to genetic factors, accounting for 10% to 15% of cases, such as FAP and Gardner syndrome.^[22]^ Of the 14 abdominal wall DT cases we reviewed, 4 were found to have FAP or Gardner syndrome. Regarding treatment and prognosis, similar to the case we report, most patients who received surgical treatment exhibited no recurrence during the short-term follow-up period.

**Table 2:** An overview on selected cases of abdominal wall DT published between 2019 and 2024 showing variable clinical behavior and different approaches in management.

Case No.	Age (yr)	Gender	Surgical/Trauma history	Pregnancy	Diagnostic	Therapy	FAP/Gardner Syndrome	Prognosis	Reference	Year
1	29	Female	PC, Colostomy	Yes	US, MRI, Biopsy	Chemotherapy	Yes	Recovered	Zubor et al^[9]^	2024
2	20s	Female	CS	No	Biopsy, CT	Chemotherapy, Surgery, Radiotherapy	NA	Recovered	Dhivakar et al^[10]^	2023
3	37	Female	None	No	CT	Surgery(R0)	NA	Recovered	Chen et al^[11]^	2023
4	60s	Female	Repair of idiopathic scoliosis, BM	No	CT,US	Surgery(R0), NSAIDs	NA	Recovered	Bracamonte et al^[12]^	2022
5	43	Female	Abdominoplasty, Hysterectomy, APP	No	CT,US,MRI	Surgery(R0)	NA	Recovered	Choe et al^[13]^	2022
6	20s	Female	Abdominoplasty	Yes	CT	Surgery	Yes	Recovered	Mohd Sulaiman et al^[14]^	2022
7	60	Male	Gastric cancer surgery	-	CT,US	Surgery(R0)	NA	Recovered	Zhang et al^[15]^	2021
8	46	Female	None	No	MRI	Surgery(R0)	No	Recovered	Loukil et al^[16]^	2021
9	30	Female	None	No	CT, MRI, Biopsy	Surgery(R0)	None	Recovered	Erdogan et al^[17]^	2021
10	33	Male	APP, CCY, Colonpolypectomy, laparotomy	-	CT, Biopsy	Surgery	Yes	Recovered	Liu et al^[18]^	2021
11	41	Female	CS	No	CT,MRI	Surgery	NA	Recovered	Mabrouk et al^[19]^	2021
12	25	Female	CO	No	MRI	RFA, Antioestrogen therapy, Surgery	Yes	Recovered	Patel et al^[20]^	2021
13	35	Female	CS	No	US, MRI	Surgery	NA	Recovered	Chen et al^[21]^	2021
14	39	Female	CS	No	US	Surgery	NA	Recovered	Chen et al^[21]^	2021

APP = appendectomy, BM = bilateral mastectomy, CCY = cholecystectomy, CO = colectomy, CS = cesarean section, CT = computed tomography, MRI = magnetic resonance imaging, NA = not available, NSAIDs = nonsteroidal anti-inflammatory drugs, PC = proctocolectomy, RFA = radiofrequency ablation, US = ultrasound.

The unpredictable course of abdominal wall DT and the uncertainty of treatment outcomes have impeded consensus on the optimal treatment approach. For slowly growing, asymptomatic cases, active surveillance (AS) may be adopted as an initial treatment strategy, involving monitoring via MRI.^[23]^ Surgical intervention should be considered for abdominal wall DT that rapidly enlarge, present symptoms that impair quality of life, remain diagnostically uncertain to the extent that malignancy cannot be excluded, or when there is a strong patient preference for surgery.^[24]^ Additionally, for inoperable or recurrent abdominal wall DT, treatment options such as radiotherapy, chemotherapy, targeted drug therapies, hormonal treatments, and nonanti-inflammatory drugs may be considered. Reports indicate that approximately one-third of patients under AS eventually require other active treatment modalities.^[25]^ In present case, the patient experienced recent progressive enlargement of a rectus abdominis DT, which led to significant abdominal pain. The patient declined further observation and strongly requested surgical intervention. After discussion in the MDT, we decided to proceed with a radical excision of the tumor. During the surgery, it was observed that the DT was completely encased by the rectus abdominis. We performed a complete excision of the DT, the involved rectus abdominis, and part of the posterior sheath. The removal of the DT resulted in a defect in the abdominal wall located in the midline area, which was successfully reconstructed using a prosthetic mesh to reinforce the area. Additionally, in cases where the defect is too large to close directly, bridging repair or component separation techniques may be employed to achieve closure, followed by reinforcement with a patch to ensure effective restoration of the abdominal wall’s structure and function. The overall local recurrence rate after DT resection stands at about 40%, primarily due to inadequate resection margins.^[26]^ Research shows that adjuvant therapies such as radiotherapy or chemotherapy are not necessary following an R0 resection of DT.^[24]^ In present case, the patient underwent an R0 resection, which successfully alleviated the abdominal pain. No adjuvant treatments were administered. Six months postoperatively, the follow-up examination showed no signs of recurrence. When the DT is too large, preoperative adjuvant therapy including chemotherapy and hormone treatments can reduce the size, thereby facilitating an R0 resection during subsequent surgery.^[27]^ In conclusion, the decision to administer preoperative and postoperative adjuvant therapies should be determined based on the specific circumstances of the patient and decided through discussions by a MDT.

The pathogenesis of DT remains elusive, but studies have shown that activation of the Wnt/β-catenin pathway is critical in DT development.^[28]^ Patients with sporadic DT present a *CTNNB1* mutation (encoding β-catenin), most frequently at mutation sites *T41A, S45F*, and *S45P*.^[29]^ The *CTNNB1* mutation prevents β-catenin protein phosphorylation, blocking its proteasomal degradation in the cytoplasm. This results in the accumulation of β-catenin, which translocates to the nucleus and forms a complex with TCF/LEF, elevating target genes such as cyclin D1 and c-Myc to foster cell proliferation and differentiation.^[28]^ DT associated with FAP typically features an *APC* mutation, which impedes β-catenin phosphorylation, leading to increased cytoplasmic β-catenin and heightened cell proliferation.^[30]^ It is important to note that *CTNNB1* mutation and *APC* mutation do not coexist in DT. The International Desmoid Tumor Working Group vigorously endorses genetic testing analysis of pathological specimens for DT to improve diagnostic accuracy and prognosis evaluation.^[24]^ Unfortunately, genetic analyses of postoperative pathological specimens are rarely reported in current case studies on DT.

Studies indicate that the *CTNNB1 S45F* mutation may be a critical prognostic factor during the AS, as a prospective study showed that the *S45F* mutation is markedly associated with tumor progression (HR = 6.24, 95% CI: 1.92–20.30), with patients harboring the *S45F* mutation in DT facing a heightened risk of requiring active treatment during AS.^[31]^ Additionally, *CTNNB1* mutations have a predictive role in postoperative recurrence, with one study indicating that sporadic DT with the *S45F* mutation tend to have a higher recurrence rate after surgery compared to those with *T41A* or wild-type mutations, and tumors harboring *S45F* and *S45P* mutations were found to be larger compared to those with *T41A* and wild-type mutations.^[32]^ Mutation analysis of the pathological specimen from present case revealed a *CTNNB1* mutation at c.121A > G, p.Thr41Ala in the E3 region with a variant frequency of 18.5%, while no *APC* mutations were detected and a colonoscopic examination did not suggest the presence of FAP, thereby corroborating that *CTNNB1* and *APC* gene mutations do not coexist in DT. Compared to *CTNNB1 S45F* mutations, our patient shows a better prognosis. We also identified a *CCND1* mutation (encoding cyclin D1) at c.723G > A in the E4 region, with a variant frequency of 100%, indicating that almost all tumor cells carry this mutation. The *CCND1* gene, through its product cyclin D1, regulates the cell cycle. Research indicates a correlation between cyclin D1 overexpression and *CTNNB1* mutation in DT.^[33,34]^ Given the rapid progression of DT in present case within a year, the *CCND1* mutation may serve as another important biomarker for the progression of abdominal wall DT. Additionally, we found higher variant frequencies in the *CYP3A4, SLIT1, RRM1, STIM1, ESR2*, and *UGT1A1* genes. However, further confirmation through basic research and large-scale clinical studies is needed to elucidate the mechanisms and clinical significance of these gene mutations in the progression of abdominal wall DT.

## 4. Conclusion

Abdominal wall DT are a rare type of aggressive and highly recurrent soft tissue tumor. Pathological examinations, including genetic testing, are crucial for assessing the clinical status of abdominal wall DT and selecting appropriate treatment strategies. Discussions within a MDT are fundamental in developing individualized treatment plans for abdominal wall DT.

We have once again demonstrated the significant clinical importance of the *CTNNB1* mutation in the diagnosis and progression of abdominal wall DT. Additionally, genes such as *CCND1, CYP3A4, SLIT1, RRM1, STIM1, ESR2*, and *UGT1A1* may also be closely related to the progression of abdominal wall DT. Future research should further explore and systematically assess the precise impact of these genetic mutations on the treatment options and prognosis of abdominal wall DT, to better guide patient management and treatment decisions.

## Acknowledgments

We acknowledge the genetic testing services provided by SurExam Bio-Tech Co.

## Author contributions

**Methodology:** Hong-Peng Guo, He Zhang, Cheng-Lin Sun.

**Writing – original draft:** Hong-Peng Guo, He Zhang.

**Writing – review & editing:** Hong-Peng Guo, He Zhang.

**Investigation:** You Li, Xing-He Pan, Jun-Jie Zhang, Cheng-Lin Sun.

**Conceptualization:** Jun-Jie Zhang, Cheng-Lin Sun.

**Project administration:** Jun-Jie Zhang.

**Resources:** Jun-Jie Zhang.

**Formal analysis:** Cheng-Lin Sun.

**Funding acquisition:** Cheng-Lin Sun.
